# A Tool Integrated into the Electronic Health Record to Guide Proper Decision-Making Regarding Peri-Endoscopic Anticoagulant Management: A Retrospective Cohort Study

**DOI:** 10.3390/jcm13175194

**Published:** 2024-09-01

**Authors:** Anja Plender, Suzanne E. Graumans, Eric Gielisse, Carlinda Bresser-de Ruyter, Simone Sissing, Marjan C. Ruiter-Jakobs, Arian Wals, Laura M. Faber

**Affiliations:** 1 Department of Internal Medicine, Red Cross Hospital, Vondellaan 13, 1942 LE Beverwijk, The Netherlands; 2 Department of Gastro-Enterology, Red Cross Hospital, Vondellaan 13, 1942 LE Beverwijk, The Netherlands; 3 Department of Research Internal Medicine, Red Cross Hospital, Vondellaan 13, 1942 LE Beverwijk, The Netherlands; cbresser@rkz.nl (C.B.-d.R.); ssissing@rkz.nl (S.S.); 4 Department of Quality & Patient Safety, Red Cross Hospital, Vondellaan 13, 1942 LE Beverwijk, The Netherlands; mruiter@rkz.nl; 5 Department of ICT Application Management, Red Cross Hospital, Vondellaan 13, 1942 LE Beverwijk, The Netherlands; awals@rkz.nl; 6 Department of Hematology, Red Cross Hospital, Vondellaan 13, 1942 LE Beverwijk, The Netherlands

**Keywords:** anticoagulation management, post-endoscopic bleeding, post-endoscopic thrombosis, decision-making tool

## Abstract

**Background**—Anticoagulants, such as vitamin-K antagonists (VKA) and direct oral anticoagulants (DOAC), are widely used among patients who undergo endoscopic procedures. To balance between bleeding and thromboembolic risks, careful decisions must be made about whether and for how long anticoagulants have to be stopped peri-endoscopically and if bridging is necessary. We created a tool in the electronic health records system (EHR) HIX (Microsoft) for invasive procedures to aid this decision-making. By selecting the anticoagulant indication or thrombo-embolic risk and the bleeding risk of the procedure, the tool automatically generates advice for periprocedural anticoagulant management. **Objectives**—This study assesses whether the tool is used properly peri-endoscopically. Secondly, it examines how many bleeding and thromboembolic events have occurred since the implementation of the tool. **Methods**—This retrospective study included all orders placed for endoscopies for patients using VKA or DOAC between 2018 and 2021. **Results**—In total, 986 endoscopies were included for analysis. In 89%, the tool was used correctly; the main error was selecting the wrong bleeding risk (7.5%). The cumulative incidence for moderate or severe bleeding events for DOAC and VKA was 2 (0.5%) and 0, respectively. The cumulative incidence of thromboembolic events for DOAC and VKA was 1 (0.2%) for each. **Conclusions**—This study evaluates the use of an EHR-integrated decision-making tool to aid peri-endoscopic anticoagulant management. By analysing the usage of the tool, we formulated several suggestions to improve the tool. Although this study is not a comparative one, we can conclude that the thromboembolic and major bleeding risks were low.

## 1. Introduction

Anticoagulants, such as vitamin K antagonists (VKA) and direct oral anticoagulants (DOAC), are widely used in the Netherlands [1]. When these patients undergo endoscopic procedures, careful decisions have to be made about whether and for how long anticoagulants have to be stopped peri-endoscopically and, for VKA users, if bridging with low-molecular-weight heparin (LMWH) is necessary [2,3].

Previous studies have shown that anticoagulants were involved in more than 6.3% of preventable medicine-related hospital admissions in the Netherlands, especially periprocedural anticoagulant use, which was found to be an important risk factor [4,5]. This is supported by a study conducted among five Dutch hospitals, which found that the local protocol regarding peri-procedural anticoagulant management was not followed in a remarkably high percentage of 11% [6].

**Figure 1:**
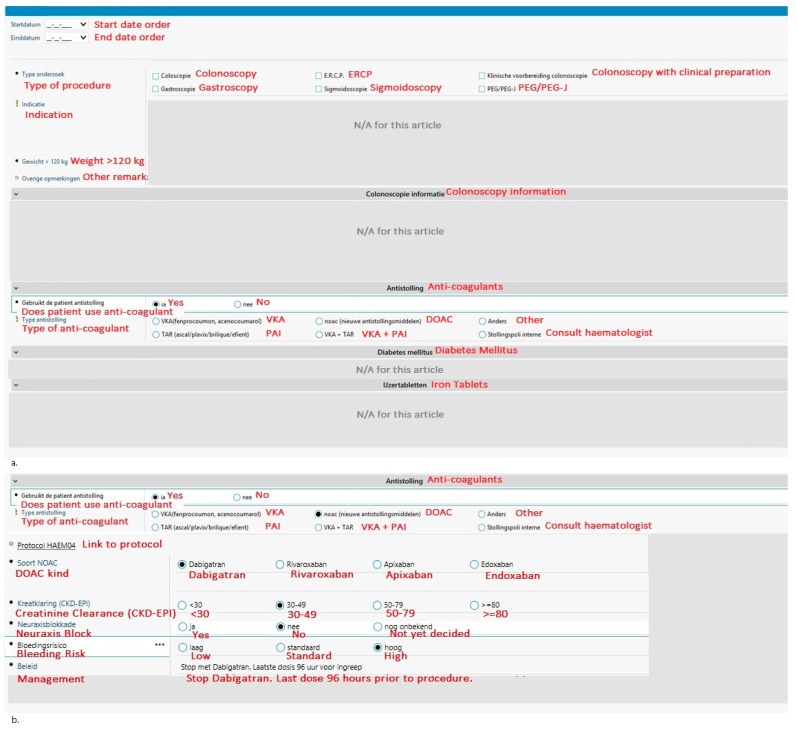

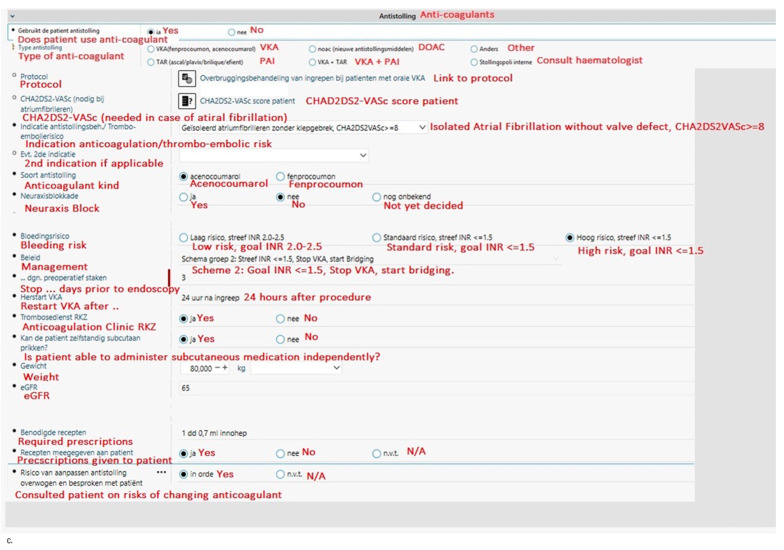
An overview of the integrated tool. The parts that are irrelevant for this article (i.e., patient characteristics and iron tablets or diabetic medication usage) have been obscured. The original tool is in Dutch; for the purpose of this article, it has been translated to English (in red). (**a**) The opening screen requires the physician to choose the procedure, the anticoagulant, and some additional important information. (**b**) If DOAC is selected in the first screen, the physician is guided through additional questions. It will then recommend periendoscopic anticoagulant management. (**c**) A similar guided drop-down menu is designed when VKA is selected; however, more additional information is required compared to the DOAC orderset before appropriate management can be generated. Abbreviations: ERCP, endoscopic retrograde cholangiopancreatography; PEG, percutaneous endoscopic gastrostomy; VKA, vitamin K antagonist; DOAC, direct-acting oral anticoagulants; PAI, plasminogen activator inhibitor; INR, international normalised ratio; RKZ, Rode Kruis Ziekenhuis (Red Cross Hospital); eGFR, estimated glomerular filtration rate.

With the aim to guide physicians in making evidence-based decisions regarding peri-endoscopic anticoagulant use and to reduce preventable complications, a tool integrated into the electronic health records system (EHR) HIX (Chipsoft) was created in 2014 at the Red Cross Hospital (Beverwijk, The Netherlands). At the time of writing, the Red Cross Hospital is the only Dutch hospital to have an EHR integrated tool such as this. The tool (see Figure 1) is integrated into the digital request forms, which are referred to as odersets in HIX, for endoscopies and surgical procedures [7]. Physicians have to select the bleeding risk of the procedure (low, intermediate, or high) for both VKA and DOAC to see if the anticoagulant should be stopped; for VKA’s, the indication or thrombo-embolic risk is also required to determine if bridging is necessary. To help physicians choose the correct thromboembolic and bleeding risks, the thromboembolic and bleeding risks corresponding to each situation are given in pop-ups (Table 1). The tool automatically generates advice for periprocedural anticoagulant management based on national guidelines published by the Dutch Internist Society (NIV) [3]. Thus, guiding physicians in making evidence-based decisions regarding peri-endoscopic anticoagulant management and saving their precious time looking for the corresponding protocol.

The present study has two goals. Primarily, it aims to assess whether the tool is used properly and safely in accordance with Dutch national guidelines. Secondly, it aims to gauge the number of thromboembolic and bleeding complications that have occurred since the implementation of the tool.

## 2. Materials and Methods

### 2.1. Research Design

This research is a retrospective and single-centre study.

### 2.2. Creation and Implementation of the Decision-Making Tool

Our decision-making tool was created in 2013 by L.M. Faber (haematologist), A. Wals (ICT), and M. Ruiter (quality control department), all authors of this article. The tool was integrated into HIX (Chipsoft), which is the EHR of this hospital, and was launched in 2014. From this moment on, the use of this tool was required to request or “order” an endoscopy for all patients. The antithrombotic guidelines of the Dutch Internist Society (NIV) [3], which were used to create the decision tool, are very comparable to international guidelines [9,10,11]; the main differences are the total duration of the VKA stop and the moment to restart the VKA. Generally, acenocoumarol is paused three days prior to endoscopy, while guidelines differ in their suggestions for fenprocoumon. Some suggest stopping seven to five days prior to the endoscopy versus the NIV guideline, which suggests pausing three to two days prior to the endoscopy with a standard dose of vitamin K (10 mg) on respectively day −3 or day −2. The current NIV guideline recommends restarting the VKA 1 day post endoscopy, while other guidelines might suggest restarting the VKA on the same day the endoscopy took place. The reason for restarting the VKA on the same day as the endoscopy is that the optimal anti-thrombotic effect of a VKA is only reached approximately 5 days post-startup. In the near future, the revised NIV guidelines will, for this reason, probably recommend restarting VKA on the same day as the endoscopy, but only after procedures with intermediate bleeding risk and not with those with a high bleeding risk. For DOAC, the optimal anti-thrombotic effect is generally reached 4–5 h post-first dose, so the restart will remain on day +1 in the NIV guidelines.

### 2.3. Research Setting

All orders made by physicians for endoscopies between the 1 January 2018 and the 1 January 2021 at the Red Cross Hospital, Beverwijk, The Netherlands, were included when a patient used either a DOAC or a VKA and did not combine this with a platelet aggregation inhibitor. Patients using a VKA were also referred to the Anticoagulation Clinic for advice regarding peri-endoscopic management in accordance with the hospital’s protocol. Emergency endoscopies were not included because no orders in the EHR are made in an emergency setting. Furthermore, orders for endoscopies of patients who had a previous endoscopy in the last 30 days were excluded. All orders of endoscopies that were cancelled were excluded.

### 2.4. Statistical Analysis

All analyses were conducted with IBM SPSS Statistics version 26 (IBM Corporation, Armonk, NY, USA). Descriptive analyses in percentages were performed. Missing data is described as such in this article.

### 2.5. Definitions

For this study, endoscopy is defined as upper gastrointestinal endoscopy, endoscopic retrograde cholangiopancreatography (ERCP), colonoscopy, or sigmoidoscopy. Endoscopies with a low bleeding risk are defined according to national protocol as diagnostic endoscopies (including those with biopsies), ERCP without sphincterotomy, endoscopies for placing stents without dilatation, and endoscopies for radiofrequency ablation for Barett’s oesophagus [3]. Endoscopies with high bleeding risk are defined according to national protocol as endoscopies with polypectomies, mucosal resection, endoscopic submucosal dissection, myotomy, submucosal tunnelling endoscopic resection, sphincterotomy, percutaneous endoscopic gastrostomy, coagulation, therapeutic double-balloon, dilatation of the oesophagus, ampullectomy, or endoscopies to treat varices or haemorrhoids [3]. In general, this means gastroscopies and sigmoidoscopies without polypectomy are considered low bleeding risk, while colonoscopies and ERCPs are considered high bleeding risk. Colonoscopies are considered high-risk because the endoscopist should be able to perform polypectomies if needed without rescheduling.

A complication is defined as a bleeding or thromboembolic event within thirty days after the endoscopic procedure. For thromboembolic events, follow-up started at the time of anticoagulation interruption, whereas for bleeding events, follow-up started at the time of the procedure. A thromboembolic event is defined as an ischaemic stroke, transient ischaemic attack (TIA), systemic embolism, acute myocardial infarction (MI), deep vein thrombosis (DVT), pulmonary embolism (PE), or fatal thromboembolism. A bleeding event is defined as any gastrointestinal bleeding. The severity of post-endoscopic bleedings is classified using the grading of the Dutch Registration of Complications in Endoscopy (DRCE) [12], which was adjusted for local use; Minor bleedings are classified as needing no admission, no transfusion, and no intervention. Mild bleedings are classified as leading to a hospital admission of less than 4 days without the need for transfusions and/or intervention; Moderate bleedings are classified as leading to a hospital admission between 4 and 10 days, 0–4 transfusions, and/or an endoscopic or percutaneous intervention; Lastly, severe bleedings are classified as leading to hospital admission of more than 10 days, more than 4 transfusions, angiographic or surgical intervention, intensive care unit admission, and/or death.

## 3. Results

### 3.1. Study Population

A total of 1059 endoscopies of patients using either VKA or DOAC were registered at the Red Cross Hospital between 1 January 2018 and 1 January 2021. After exclusion, a total of 986 endoscopies were included in the final analysis (Table 2).

### 3.2. Tool Safety

For 880 (89%) of the endoscopies, the tool was used correctly to make a management plan, with a more or less even distribution over DOAC and VKA users. A total of 106 errors were made; the predominant error was selecting bleeding risk incorrectly (low, intermediate, or high) (7.5%) (Table 3). In 2.0% of endoscopies, anticoagulant management deviated from advised management by the tool; most frequently, the anticoagulant was discontinued too late (0.7%).

In 0.8% of VKA patients, the “ordering” or attending physician incorrectly intended not to bridge or incorrectly intended not to prescribe vitamin K for phenprocoumon-using patients prior to endoscopy by selecting the wrong thromboembolic risk. However, these patients received the correct anticoagulation management peri-endoscopically because these errors were corrected by the VKA dosing physicians (in the Netherlands, all patients using VKA are under the supervision of a haematologist who monitors and determines their dosing regimen; referred to as a dosing physician). In our hospital, the dosing physicians work for the anticoagulation clinic that is located inside our hospital; on a daily basis, they check the periprocedural advice generated by our decision-making tool integrated in the EHR and have the authority to overrule the generated advice. Their final advice regarding peri-endoscopic anticoagulant management can be found in the corresponding order in the EHR, which is open to the attending physician and the physician performing the endoscopy. Patients using a DOAC are not under the supervision of a dosing physician, and bridging is not required for these patients given the short half-lives of DOACs.

For 37 (3.8%) patients, physicians deviated from the protocol for medical reasons. For example, some physicians considered their patients’ thrombosis risk too high to safely discontinue anticoagulants for reasons that are not taken into account in the national protocol. Therefore, they decided to continue anticoagulants while protocol indicated otherwise; during these endoscopies, no high-bleeding-risk intervention is possible.

Table 4 shows the consequences of selecting the bleeding risk incorrectly. In 59.5%, selecting the incorrect bleeding risk had no consequences for the patients—largely because it was corrected by dosing physicians of the Anticoagulation Clinic, but also because peri-endoscopic anticoagulation management is similar for intermediate and high bleeding risk endoscopies for VKA users. However, 14.7% of the incorrectly selected bleeding risk for DOAC patients resulted in the inappropriate continuation of anticoagulation, and in another 41.2% it resulted in too-late discontinuation. In 5 out of 197 patients using dabigatran (2.5%), the eGFR was too old or missing when the physician determined peri-endoscopic anticoagulant management. In a notable 32.4%, the DOAC was unnecessarily discontinued, exposing the patients to unwarranted risks of thrombosis.

In 2 patients, an incorrectly selected bleeding risk resulted in a re-endoscopy because they required polypectomy, which could not be performed initially because they were still using their anticoagulant. Furthermore, 5 interventions were performed under anticoagulation or after anticoagulation was discontinued too late, exposing patients to a higher bleeding risk than necessary. In these 5 patients, 1 complication occurred. This patient had moderate bleeding following his initial endoscopy after his anticoagulant was discontinued too late. A clip needed to be placed at the polypectomy site to stop the bleeding, and he required a transfusion. The polyp was 12 mm. The other 4 patients did not get a bleeding complication; all their polyps were <10 mm.

### 3.3. Complications

During our inclusion period of 3 years, the incidence for moderate or severe bleeding events was 2 (0.2%) and for severe thromboembolic events, 2 (0.2%), see Table 5.

In total, 15 (1.5%) bleeding events occurred, of which 9 (2.1%) and 6 (1.1%) occurred in the DOAC and VKA groups, respectively; there was no significant difference between groups (X2 (2, N = 986) = 1.6853, *p* = 0.194222). Of these bleeding events, 2 patients needed transfusion, 7 underwent a second endoscopy for inspection and possible treatment of bleeding, and 3 were admitted for observation, but no further intervention was necessary. Another 3 had minor bleeding complications needing no intervention. Almost all bleeding events happened among patients who underwent colonoscopy (mostly with polypectomy), except for one patient who was diagnosed with a malignant stomach ulcer during gastroscopy. She had haematemesis in the days following her gastroscopy; another gastroscopy was performed, but no other endoscopic intervention was needed.

There were 2 (0.2%) thromboembolic events. One patient, who had received a biovalve 7 months prior to the colonoscopy, had a stroke. The discontinuation of dabigatran was approved by the cardiologist and conformed to the national guidelines. The other patient had a pulmonary embolism even though the VKA was not stopped for the procedure according to protocol; his international normalised ratio (INR) on the day of his gastroscopy was 3.6. A third patient had thrombophlebitis in his left upper leg 24 days after the colonoscopy, but there were no signs of DVT (Table 6).

In total, 2 complications happened within the group of incorrectly selected bleeding risk, consisting of 74 cases (2.7%), compared to 15 complications in the remaining 912 cases (1.6%). A chi-square test of independence showed that there was no significant association between selection of incorrect bleeding risk and complications (X2 (2, N = 986) = 0.4521, *p* = 0.50132). As previously stated, in 59.5% of the cases in which the incorrect bleeding risk was selected, the incorrect selection did not result in incorrect anti-coagulant management, which could explain why there is no significant difference.

## 4. Discussion

This study evaluates the use of an EHR-integrated decision-making tool to aid peri-endoscopic anticoagulant management for VKA and DOAC-using patients. To our knowledge, this is the first tool integrated into the EHR designed to give evidence-based, protocol-driven advice regarding peri-endoscopic anticoagulant use.

Analysis of the proper and safe use of our decision-making tool showed firstly that the tool was used incorrectly 10.7% of the time, indicating that the tool is not perfect (yet). To make the use of the tool easier and more intuitive, we have formulated several suggestions to improve the tool; see Box 1. Most importantly, as physicians most often make the mistake of choosing the wrong bleeding risk (7.5%), we suggest that the tool automatically fills in the bleeding risk based on the chosen procedure from the overview in the tool, which is based on national guidelines. When needed, it is possible for the physician to adjust this bleeding risk. Secondly, we suggest that physicians have to motivate their choice when deviating from the given advice. In this manner, we hope to reduce mistakes that could have been made by physicians who incorrectly assumed they knew the protocol by heart. Furthermore, we hope to reduce the number of delayed or second endoscopies needed because peri-endoscopic anticoagulation management was inadequate. In our study, this number was already fairly low. Possibly this is because our decision-making tool urges physicians to think about peri-endoscopic anticoagulation management, and at the same time it offers an overview of the national guidelines. Moreover, we are planning to introduce mandatory e-learnings and presentations for physicians about the tool to increase awareness and knowledge of peri-procedural anticoagulation management in accordance with national guidelines. Furthermore, we recently made a website for the physicians in our region with information on the periprocedural procedure and how to use the tool [7].

Box 1 **Recommendations to improve the decision-making tool.**

- The tool automatically selects the bleeding risk based on the chosen procedure and the national bleeding-risk protocol; this can be adjusted by the physician if needed.

- At the end of the tool, the physician ordering the endoscopy should tick a box “I’m following the advice/protocol as listed above” or tick a box “I’m not following protocol” and be obliged to motivate this in a text box.

- Introducing special e-learnings for medical specialists on this subject and improving awareness and knowledge of anticoagulation in accordance with the national guidelines

- The tool should only ask for an eGFR when this influences anticoagulation management.

The main aim of this study is to introduce the decision tool and to assess whether it is used properly and safely to conform to the Dutch national guidelines. A limitation of this study is that it does not compare the peri-endoscopic tromboembolic and bleeding risk of this cohort to a historic cohort of patients before this tool was integrated. However, we can say that both moderate or severe bleeding and thromboembolic complications (0.2% and 0.2%, respectively) were low in our study group. In the general population, including patients not using anticoagulants, haemorrhage was reported in 0.1–0.6% of patients [13]. In another study looking at anticoagulated patients, major hemorrhagic events were reported in 2% [8]. Some studies show that VKA users are at a greater risk for hemorrhagic events than DOAC users; for example, the study by Huie et al. showed that warfarin increased the risk of post-polypectomy bleeding with an odds ratio of 13.37 (95% confidence interval 4.10–43.65) [14]. We did not confirm this finding in the present study; like the study by Inoue et al., we found no significant difference between groups [15]. Many studies have reported an increased risk of bleeding associated with bridging with LMWH [8,13]. The number of bridgings in our study was low, which may be due to the use of the EHR tool.

Furthermore, the thromboembolic incidence (0.2%) seems to be low in our study population. When roughly comparing thromboembolic complications 30 days post endoscopy to a large study conducted in Hong Kong looking at patients using DOAC or warfarin, we found a lower rate in our cohort (0.2%) compared to theirs (1.0–2.4% depending on the type of DOAC/vitamin K antagonist) [16]. However, study populations between these studies vary too much to draw conclusions.

The present study solely looked at the effect of the implementation of the tool regarding VKA and DOAC, while the use of platelet aggregation inhibitors (for instance, aspirin, clopidogrel, prasugrel, and ticagrelor) peri-endoscopically might also benefit from guided decision-making through the tool [2,3]. At the hospital, the tool is being trialled to include platelet aggregation inhibitors, but evaluation fell outside the scope of the current study. The tool will also be optimised based on the recommendations in Box 1. Additionally, with artificial intelligence greatly improving in recent times, it is sensible to eventually integrate artificial learning in the tool to enhance its performance and its clinical benefit; we hope that EHR undergoes advances in the near future such that artificial learning can be integrated into tools similar to the one we created.

It is expected that our tool will improve evidence-based management regarding peri-endoscopic anticoagulants while saving on costs; previous studies showed that following guidelines resulted in a favourable risk ratio while driving down costs [17,18]. This monocentric study provides a basis for future research examining the effects of the implementation of this tool in other settings. Future research will assess whether the implementation of an optimised version of the tool will lead to fewer complications. Furthermore, clinicians’ satisfaction regarding this decision tool will be examined.

To summarise, this study evaluates the use of an EHR-integrated decision-making tool to aid peri-endoscopic anticoagulant management. We conclude that the tool was used properly in most cases but could benefit from some adjustments. Although the main aim of this study was not to look at thromboembolic and major bleeding risks since the implementation of the tool, we found that these were already quite low without the intended improvements.

## Figures and Tables

**Table 1:** Arterial and venous thromboembolic risk.

Risk	Annual Risk	Clinical Status
Arterial thromboembolic risk		
High	>10%	- Isolated AF, without valve dysfunction, and CHA_2_DS_2_-VASC-score of 8 or 9 ^†^; - Isolated AF with rheumatic heart disease; - AF with mechanic heart valve or recent (<6 months) CVA/TIA regardless of the CHA_2_DS_2_-VASC score; - Mechanic heart valve in mitral/tricuspidal/pulmonal position; - Prosthetic heart valve placed in the last 3 months; - Prosthetic heart valve with extra risk factor ^‡^; - Mechanic heart valve old model: Caged ball, tilting disc (Starr-Edwards, Björk–Shiley); - Intracardial thrombus.
Low	<10%	- Isolated AF and CHA_2_DS_2_-VASC-score 0–7 ^†^; - Mechanic heart valve in aortic position without extra risk factors ^‡^; - Reoccurring CVA/TIA without cardioembolic source - No more than one TIA or CVA
Venous thromboembolic risk		
High	>10%	- <3 months after first/reoccurring VTE;
Low	<10%	- 3 or more months after first/reoccurring VTE;

Table derived from the national guideline from the Dutch Internist Society (NIV), “Guideline Antithrombotic policy,” page 273 [3]. ^†^ CHA_2_DS_2_-VASC-score can be calculated using http://eurheartj.oxfordjournals.org/content/33/21/2719.full.pdf (accessed on 2 April 2024) [8]. ^‡^ Risk factors are: AF, left ventricular ejection fraction < 35%, medical history of thromboembolism. Abbreviations: <, less than; >, greater than; ≥, greater than or equal to; AF, atrial fibrillation; CVA, cerebrovascular accident; TIA, transient ischemic attack; VTE, venous thromboembolism.

**Table 2:** Patient Characteristics.

	Total (n = 986)	DOAC (n = 429)	VKA (n = 557)
Age, median (IQR)	73.1 (67.6–78.1)	71.7 (66.6–75.4)	74.1 (68.9–79.5)
ASA-score, median (IQR)	2 (2–2)	2 (2–2)	2 (2–2)
Gender, n (%)			
- Female	412 (41.8)	170 (39.6)	242 (43.4)
- Male	574 (58.2)	259 (60.4)	315 (56.6)
Investigation, n (%)			
- Gastroscopy	182 (18.4)	73 (17.0)	109 (19.6)
- ERCP	16 (1.6)	6 (1.4)	10 (1.8)
- Colonoscopy	696 (70.6)	315 (73.4)	381 (68.4)
- Sigmoidoscopy	32 (3.0)	13 (2.8)	19 (3.4)
- Colonoscopy + Gastroscopy	59 (6.0)	22 (5.1)	37 (6.6)
- Gastroscopy + Sigmoidoscopy	1 (0.1)	0 (0.0)	1 (0.2)
Procedure, n (%)			
- Polypectomy	366 (37.1)	167 (38.9)	199 (35.7)
- Biopsy	159 (16.1)	61 (14.2)	98 (17.6)
- Polypectomy + Biopsy	29 (2.9)	9 (2.1)	20 (3.6)
- Sphincterotomy	9 (0.9)	3 (0.7)	6 (1.1)
- EMR	8 (0.8)	3 (0.7)	5 (0.9)
- Stent	7 (0.7)	2 (0.5)	5 (0.9)
- Dilatation	7 (0.7)	6 (1.4)	1 (0.2)
- APC	4 (0.4)	2 (0.5)	2 (0.4)
- eFTR	1 (0.1)	1 (0.2)	0 (0)

Abbreviations: APC, Argon plasma coagulation; ASA-score, American Society of Anesthesiology score; DOAC, direct oral anticoagulant; eFTR, Endoscopic full thickness resection; EMR, endoscopic mucosal resection; ERCP, Endoscopic retrograde cholangiopancreatography; IQR, interquartile range; n, number; VKA, vitamin K antagonist.

**Table 3:** Errors made by physicians using the decision-making tool.

	Total (n = 986)	DOAC (n = 429)	VKA (n = 557)
Total errors, n ^†^	106	51	55
Errors in filling in tool, n (%)			
- Incorrect bleeding risk selected ^‡^	74 (7.5)	34 (7.9)	40 (7.2)
- Incorrect type of anticoagulant selected	5 (0.5)	4 (0.9)	1 (0.2)
- eGFR unknown/incorrectly filled in (DOAC) ^§^	4 (0.4)	4 (0.9)	N/A
- Incorrect thrombo-embolic risk selected (VKA) ^¶^	3 (0.3)	N/A	3 (0.5)
Errors in following suggested management by tool, n (%)			
- Late discontinuation anticoagulant	7 (0.7)	7 (1.6)	0 (0)
- Early discontinuation anticoagulant	2 (0.2)	2 (0.5)	0 (0)
- Unclear communication with patient	4 (0.4)	0 (0)	4 (0.7)
- Unknown INR on day of endoscopy (VKA) ^¶^	3 (0.3)	N/A	3 (0.5)
- No bridging with LMWH when should have (VKA) ^¶^	2 (0.2)	N/A	2 (0.4)
- No vitamin K (phenprocoumon patients) (VKA) ^¶^	2 (0.2)	N/A	2 (0.4)
Deviated from protocol for medical reasons, n (%)	37 (3.8)	13 (3.0)	24 (4.3)

^†^ In two cases two different type of errors were made using the decision-making tool, so in 104 cases the decision-making tool was used incorrectly but in total 106 errors were made. ^‡^ Consequences of selecting the incorrect bleeding risk is shown in Table 3. § Relevant for correct usage of dabigatran. ^¶^ Errors in management plan made by physicians were corrected before endoscopy took place by special vitamin K antagonists dosing physicians of the Anticoagulation Clinic. Abbreviations: DOAC, direct oral anticoagulant; eGFR, estimated glomerular filtration rate; INR, international normalized ratio; LMWH, low-molecular-weight heparin; n, number; N/A, not applicable; VKA, vitamin K antagonist.

**Table 4:** Consequences of incorrect bleeding risk selected.

	Total (n = 74)	DOAC (n = 34)	VKA (n = 40)
Direct consequences, n (%)			
- Anticoagulants not discontinued	5 (6.8)	5 (14.7)	0 (0)
- Anticoagulants discontinued too late	14 (18.9)	14 (41.2)	0 (0)
- Anticoagulants unnecessary discontinued	11 (14.9)	11 (32.4)	0 (0)
- Anticoagulants used according to protocol ^†^	44 (59.5)	4 (11.8)	40 (100)
Indirect consequences, n (%)			
- Re-endoscopy	2 (2.7)	2 (5.9)	0 (0)
- Polypectomy under anticoagulation ^‡^	5 (6.8)	5 (14.7)	0 (0)

^†^ All orders for VKAs were altered by dosing physicians. ^‡^ All polyps where <10 mm except for one, which was 12 mm. This patient had a moderate bleeding complication after anticoagulant was discontinued too late, for more details see Table 6, patient 10. No other complications occurred in this group. 4 patients discontinued late, only one patient did not discontinue anticoagulant at all (polyp size was 5 mm). Abbreviations: DOAC, direct oral anticoagulant; VKA, vitamin K antagonist; n, number. Most endoscopies were performed as planned, if endoscopist encountered polyps they either rescheduled or did polypectomy regardless.

**Table 5:** Overall complications since introduction of the decision-making tool.

	Total (n = 986)	DOAC (n= 429)	VKA (n = 557)
Complication, n (%) ^†^	17 (1.7)	10 (2.3)	7 (1.3)
- Bleeding event, n (%) ^‡^	15 (1.5)	9 (2.1)	6 (1.1)
+ minor	3 (0.3)	1 (0.2)	2 (0.4)
+ mild	10 (1.0)	6 (1.4)	4 (0.7)
+ moderate	2 (0.2)	2 (0.5)	0 (0)
+ severe	0 (0)	0 (0)	0 (0)
- Thromboembolic event, n (%) ^§^	2 (0.2)	1 (0.2)	1 (0.2)

^†^ Defined as a bleeding or thromboembolic event within thirty days after the endoscopic procedure. ^‡^ Grading of severity for post-procedural bleeding according to Dutch Registration of Complications in Endoscopy (DRCE): Minor bleeding: no admission, no transfusion and no intervention needed; Mild bleeding: admission <4 days; Moderate bleeding: admission of 4–10 days, 0–4 transfusions and/or endoscopic or percutaneous intervention; Severe bleeding: admission >10 days, >4 transfusions, angiographic or surgical intervention, intensive care unit admission and/or death. ^§^ Defined as an ischemic stroke, transient ischemic attack, systemic embolism, acute myocardial infarction, deep vein thrombosis, pulmonary embolism or fatal thromboembolism. Abbreviations: DOAC, direct oral anticoagulant; n, number; VKA, vitamin K antagonist.

**Table 6:** Elaborate characteristics of bleeding and thrombotic complications.

Pt No.	Sex	Age	Gastro-Intestinal Malignancy	DOAC or VKA	Anti-Coagulant Variety	Procedure	Complication	Severity of Bleeding Event	Severity of Thrombotic Event	INR	Bridged	ASA-Score	Inter-Vention	Number of Polyps Removed	Location of Polyp(s)	Polyp Size † (in mm)	Number of Biopsies	Bleeding during Scopy
**1**	M	69	No	DOAC	Edoxaban	Colono-scopy	Rectal bleeding, no intervention needed.	Minor	N/A	N/A	N/A	2	Polyp-ectomy	13	Entire colon	2–10	0	No
**2**	M	80	Yes	DOAC	Rivaroxaban	Colono-scopy + Gastro-scopy	Rectal bleeding, admission for observation.	Mild	N/A	N/A	N/A	2	Polyp-ectomy + biopsies	5	Cecum, ascen-dens, sigmoid-eum, rectum	4–10	1	No
**3**	F	75	No	DOAC	Dabigatran	Colono-scopy	Rectal bleeding, was admitted, underwent rescopy and received blood transfusion and ferinject.	Moder-ate	N/A	N/A	N/A	3	Polyp-ectomy	1	Ascen-dens	20	0	No
**4**	F	72	No	DOAC	Apixaban	Colono-scopy	Rectal bleeding, was admitted and underwent rescopy.	Mild	N/A	N/A	N/A	2	Polyp-ectomy	6	Ascen-dens, transver-sum, descen-dens	3–10	0	No
**5**	M	63	No	DOAC	Dabigatran	Colono-scopy	Rectal bleeding, was admitted and underwent rescopy.	Mild	N/A	N/A	N/A	2	Polyp-ectomy	2	Sigmoid-eum, rectum	2–15	0	No
**6**	F	68	No	DOAC	Dabigatran	Colono-scopy	Rectal bleeding, admission for observation.	Mild	N/A	N/A	N/A	2	Polyp-ectomy	2	Ascen-dens	2–8	0	No
**7**	F	68	No	DOAC	Dabigatran	Colono-scopy	Stroke, received biovalve 7 months prior, discontinuation was approved by cardiologist.	N/A	Severe	N/A	N/A	3	None	0	N/A	N/A	0	No
**8**	F	67	No	DOAC	Rivaroxaban	Colono-scopy	Rectal bleeding, admission for observation.	Mild	N/A	N/A	N/A	2	Polypec-tomy	2	Ascen-dens, sigmoid-eum	3–23	0	No
**9**	M	61	No	DOAC	Dabigatran	Colono-scopy	Rectal bleeding, was admitted and underwent rescopy.	Mild	N/A	N/A	N/A	2	Polyp-ectomy	5	Ascen-dens, sigmoid-eum	1–25	0	No
**10**	V	81	No	DOAC	Dabigatran	Colono-scopy + Gastro-scopy	Rectal bleeding, was admitted, underwent rescopy and received blood transfusion. Anticoagulant was discontinued too late.	Moderate	N/A	N/A	N/A	2	Polyp-ectomy	5	Entire colon	4–12	0	No
**11**	M	72	No	VKA	Aceno-coumarol	Colonos-copy	Rectal bleeding, was admitted and underwent rescopy.	Mild	N/A	1.1	Yes	3	EMR	1	Rectum	40	0	Yes
**12**	M	70	No	VKA	Aceno-coumarol	Gastro-scopy	Pulmonary embolism, acenocoumarol was discontinued for one day, INR on day of gastroscopy was 3.6.	N/A	Severe	3.6	No	?	None	0	N/A	N/A	0	No
**13**	F	85	Yes	VKA	Aceno-coumarol	Gastro-scopy	Hematemesis, malignant ulcer in situ, underwent rescopy.	Mild	N/A	?	No	2	Biopsies	0	N/A	N/A	5	No
**14**	M	81	No	VKA	Aceno-coumarol	Colono-scopy	Rectal bleeding, was admitted and underwent rescopy.	Mild	N/A	1.2	No	2	Polyp-ectomy + biopsies	18	Entire colon	<10–20	10	No
**15**	F	71	No	VKA	Aceno-coumarol	Colono-scopy	Rectal bleeding, was admitted and underwent rescopy.	Mild	N/A	1.1	No	2	EMR	2	Ascen-dends	15–20	0	No
**16**	M	73	No	VKA	Aceno-coumarol	Colono-scopy	Rectal bleeding, no intervention needed.	Minor	N/A	?	No	2	Polyp-ectomy	5	Cecum, ascen-dens, sigmoid-eum	<5–15	0	No
**17**	F	79	Yes	VKA	Aceno-coumarol	Colono-scopy + Gastro-scopy	Rectal bleeding, no intervention needed.	Minor	N/A	1.2	No	2	Biopsies	0	N/A	N/A	5	No

^†^ if multiple polypectomies were performed range of polyp size is given. Abbreviations: Pt No, patient number; ?, unknown at time of endoscopy or not documented in electronic health record; ASA, American Society of Anaesthesiology score; DOAC, direct oral anticoagulant; EMR, endoscopic mucosal resection; F, female; INR, international normalized ratio; M, male; mm, millimeter; N/A, not applicable; VKA, vitamin K antagonist.

## Data Availability

Data supporting this study cannot be made available due to privacy of patients.
